# Structural brain imaging correlates of ASD and ADHD across the lifespan: a hypothesis-generating review on developmental ASD–ADHD subtypes

**DOI:** 10.1007/s00702-016-1651-1

**Published:** 2016-12-21

**Authors:** Nanda Rommelse, Jan K. Buitelaar, Catharina A. Hartman

**Affiliations:** 1 Department of Psychiatry, Donders Institute for Brain, Cognition and Behavior, Radboud University Medical Center, Nijmegen, The Netherlands; 2 Karakter, Child and Adolescent Psychiatry University Center, Nijmegen, The Netherlands; 3 Department of Cognitive Neuroscience, Donders Institute for Brain, Cognition and Behavior, Radboud University Medical Center, Nijmegen, The Netherlands; 4 Department of Psychiatry, Interdisciplinary Center of Psychopathology and Emotion Regulation (ICPE) & Research School of Behavioral and Cognitive Neuroscience, University Medical Center Groningen, University of Groningen, Groningen, The Netherlands

**Keywords:** Autism spectrum disorder, Attention-deficit/hyperactivity disorder, Comorbidity, Life span, MRI, Brain, Adults, Adolescence, Anterior cingulate cortex, Prefrontal

## Abstract

We hypothesize that it is plausible that biologically distinct developmental ASD–ADHD subtypes are present, each characterized by a distinct time of onset of symptoms, progression and combination of symptoms. The aim of the present narrative review was to explore if structural brain imaging studies may shed light on key brain areas that are linked to both ASD and ADHD symptoms and undergo significant changes during development. These findings may possibly pinpoint to brain mechanisms underlying differential developmental ASD–ADHD subtypes. To this end we brought together the literature on ASD and ADHD structural brain imaging symptoms and particularly highlight the adolescent years and beyond. Findings indicate that the vast majority of existing MRI studies has been cross-sectional and conducted in children, and sometimes did include adolescents as well, but without explicitly documenting on this age group. MRI studies documenting on age effects in adults with ASD and/or ADHD are rare, and if age is taken into account, only linear effects are examined. Data from various studies suggest that a crucial distinctive feature underlying different developmental ASD–ADHD subtypes may be the differential developmental thinning patterns of the anterior cingulate cortex and related connections towards other prefrontal regions. These regions are crucial for the development of cognitive/effortful control and socio-emotional functioning, with impairments in these features as key to both ASD and ADHD.

## Background and aim

Autism spectrum disorder (ASD) and attention deficit hyperactivity disorder (ADHD) frequently co-occur. Exact comorbidity rates are not well known due to the DSM-IV restriction of diagnosing ASD and ADHD in the same individual. Hence, available diagnostic comorbidity estimates are based on only a few papers and are likely biased (see for review Rommelse et al. 2010). In addition, prevalence of the full diagnosis of ASD (and ADHD) are much more affected by administrative changes as is the case for prevalence of symptoms (Lundström et al. 2015 BMJ). Several papers have reported on the overlapping prevalence of symptoms of ASD and ADHD, a less biased method to examine the overlapping occurrence of both disorders (Lundstrom et al. 2015). A recent paper on a population-based twin sample of *N* = 17,000 children (9–12 years) concluded that ‘individuals with all three (i.e., social impairments, communication impairments, restricted repetitive behaviors) ASD domains in the sample never showed hyperactivity alone but often exhibited co-occurring impulsivity, or co-occurring impulsivity and inattention’ (p. 447; Ronald et al. 2014). Based on this sample (Table 2, p. 445; Ronald et al. 2014), 82% of the boys and 95% of the girls with high ASD traits on all three domains showed impairments on at least one of the three ADHD domains (inattention, hyperactivity, impulsivity); 42% of the boys and 62% of the girls with ASD traits on all three domains showed impairments on at least two ADHD symptom domains; and 24% of the boys and 66% of the girls with ASD traits on all three domains showed impairments on all three ADHD symptom domains. This shows that the vast majority of children with ASD (particularly girls) suffer from co-occurring ADHD traits on at least one domain.

Clinicians often struggle deciding if one, or the other, or both disorders, best describe the patient’s problems. A strong body of literature has convincingly shown that many similarities are present in genetic factors, brain characteristics, and cognitive profiles (Doherty and Owen 2014; Dougherty et al. 2016; Edlow 2016; Fluegge 2016; Hartman et al. 2016; Homberg et al. 2016; Johnson et al. 2015; Pinto et al. 2016; Polderman et al. 2014; Rommelse et al. 2010, 2011; Ronald and Hoekstra 2011; Ronald et al. 2014; Scheinost et al. 2016; Simons et al. 2016; Tan et al. 2016; Taylor et al. 2015; Visser et al. 2016). However, this body of literature is for the vast majority based on the findings in childhood samples. Despite our knowledge of change in the mechanisms underlying the development of healthy individuals until elderly age, studies on neurodevelopmental disorders like ADHD and ASD have been strongly restricted to childhood. Age of onset for ASD and ADHD is nearly always in childhood (Jensen and Steinhausen 2015; but see recent work on late-onset ADHD, Agnew-Blais et al. 2016; Caye et al. 2016; Moffitt et al. 2015), which is the probable reason for this bias in the literature. This bias is remarkable, since it is already well known that developmental changes take place in both ADHD and ASD symptom domains also after childhood.

We recently elaborately reviewed the literature on ASD–ADHD symptom development across the lifespan and related cognitive/effortful control skills (Hartman et al. 2016), and concluded that both ADHD and ASD symptom constellations are not at all stable across development—with some symptom dimensions (attention problems and social problems) being much more persistent than other symptom dimensions (hyperactivity/impulsivity and repetitive behaviors). Importantly, we argued that the co-occurrence of ADHD and ASD symptoms seem to vary with age as well, with strongest co-occurrence in adolescence and least co-occurrence in toddlerhood and old age (Hartman et al. 2016). In line with this change, cognitive/effortful control—a core component of self-regulation and functionally interdependent with social skills—undergoes substantial changes with age. Initial evidence suggests that complex executive functions (EFs), based on ratings in daily life, likewise show the greatest deviance in adolescence relative to normative development (Hartman et al. 2016). If indeed the socio-cognitive mechanisms behind the ASD–ADHD symptom co-occurrence may show changes across the lifespan as well, we argued that periods of strong cognitive and social change, i.e., infancy/toddlerhood, adolescence and old age, provide excellent time windows to study how ASD or ADHD develop side by side (Hartman et al. 2016).

Preliminary support for mechanistic change across development was found when reviewing genetically informative studies (Rommelse and Hartman 2016). Longitudinal twin studies on ADHD symptoms report that new, age specific genetic effects influence both ADHD symptom domains in adolescence and adulthood (e.g., Greven et al. 2011; Pingault et al. 2015). It is plausible that this holds for ASD as well, although there are currently no data to confirm this. Importantly, these findings pertain to heritability estimates based on quantitative genetic studies (mostly twin studies). An elucidation of the relevance of identified gene variants over the age span rests on molecular genetic studies with patients followed over the lifespan. Molecular genetic studies on ADHD and ASD are only beginning to take lifespan developmental change into account.

**Fig. 1:**
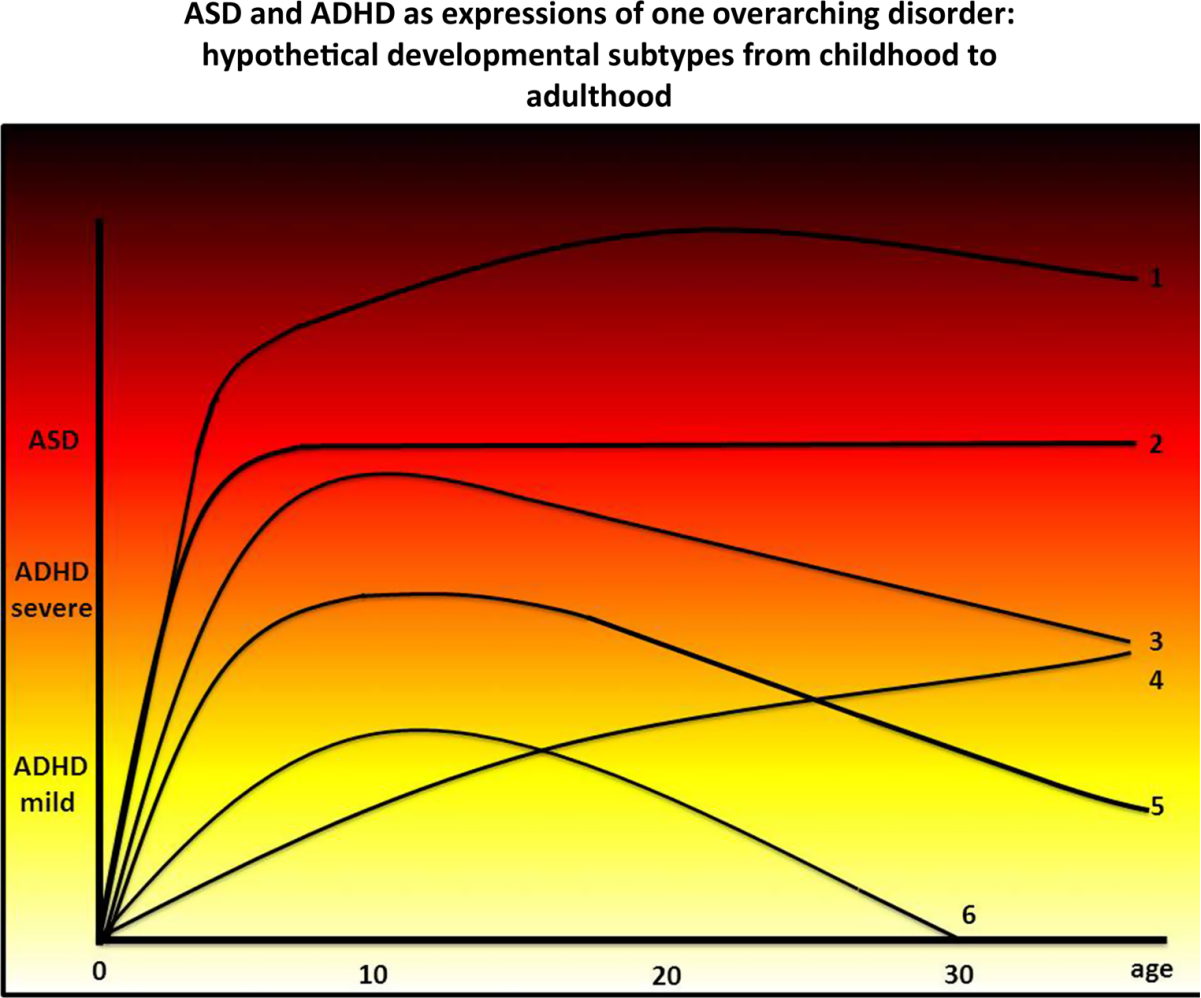
ASD and ADHD as expressions of one overarching disorder: hypothetical developmental subtypes from childhood to adulthood. Hypothetical ASD–ADHD developmental subtypes from childhood to adulthood within the framework of an overarching disorder model. From *top* to *bottom 1* a very severe developmental subtype with still increasing ASD symptoms during adolescence; *2* typically observed developmental subtype with increasing symptoms in childhood and stabilization afterwards; *3* subtype with very severe ADHD symptoms approaching ASD levels during adolescence and becoming milder afterwards; *4* recently reported late-onset ADHD subtype with symptoms increasing during adolescence; *5* typically observed developmental subtype with ADHD symptoms without ASD symptoms, with ADHD symptoms becoming milder during adolescence; *6* typically observed mild ADHD subtype that mostly does not require intervention

Thus, the above outlined results indicate changes occur in (the overlap of) ADHD and ASD symptom domains across the lifespan, which may be linked to age-related changes in involved etiological mechanisms. We hypothesize that it is plausible that biologically distinct developmental ASD–ADHD subtypes are present, each characterized by a distinct time of onset of symptoms, progression and combination of symptoms. We previously hypothesized that ASD and ADHD may be seen as different manifestations of one overarching disorder (Rommelse et al. 2011; van der Meer et al. 2012). The manifestations of this overarching disorder may range from ADHD with few if any social handicaps and problems, through ADHD with greater levels of social and communicative problems, to ASD as the most severe subtype characterized by additional and more severe social problems. Particularly during developmental periods of rapid change driven by normative genetic and environmental mechanisms (such as early childhood, adolescence, elderly age), symptoms may either become milder or reversely, increase and become fully manifest (Hartman et al. 2016). Based on the literature and clinical experience, we hypothesize that six meaningful developmental ASD–ADHD subtypes exist (Fig. 1): a very severe developmental subtype with still increasing ASD symptoms during adolescence; a typically observed developmental subtype with increasing symptoms in childhood and stabilization afterwards; a subtype with very severe ADHD symptoms approaching ASD levels during adolescence and becoming milder afterwards; a recently reported late-onset ADHD subtype with symptoms increasing during adolescence; a typically observed developmental subtype with ADHD symptoms without ASD symptoms, with ADHD symptoms becoming milder during adolescence; and a typically observed mild ADHD subtype that mostly does not require intervention. At least three predictions follow from this model:

1. If ASD is indeed best regarded as the more severe form, it is expected that infants and toddlers transgressing towards ASD will show precursors and symptoms of ADHD before the core symptoms of ASD become manifest (developmental subtypes 1, 2, 3);
2. It may be expected that in adolescence and young adulthood, when ADHD symptom improvement is most pronounced in a proportion of individuals, ASD symptoms will likely improve as well. That is, this ‘transitory’ subtype suggests that the severe form of the overarching disorder is not likely to remain in the absence of the milder ADHD symptoms (developmental subtype 3);
3. For some children, once development has derailed and the most severe form of the disorder (ASD) has become manifest, an unrepairable damage has taken place and full recovery does not take place. These individuals that have developed ASD will always remain symptomatic to some extent, with co-occurring ADHD symptoms present. This ‘scarring’ subtype finds its parallel in, for example, permanent hearing loss after recurrent ear infections, leading to permanent bone malformations in the inner ear despite the absence of recurrent ear infections (developmental subtypes 1 and 2).

These developmental ASD–ADHD subtypes may each be linked to distinct brain developmental pathways. The aim of the present narrative review was therefore to explore if structural brain imaging studies may shed light on key brain areas that are linked to both symptom constellations and undergo significant changes during development, particularly in the adolescent years and beyond. The findings may possibly pinpoint to brain mechanisms underlying the developmental ASD–ADHD subtypes. To this end we bring together the literature on ASD and ADHD structural brain imaging symptoms and particularly highlight the adolescent years and beyond. Our work will illustrate the need for a lifespan approach to detect brain-based developmental ADHD–ASD phenotypes.

## Methods

Given that the focus of empirical studies, reviews and meta-analyses are usually on childhood years, we only briefly summarized the childhood literature and focused our search in the literature on the much less studied developmental periods of adolescence and adulthood instead. Studies were included reporting on dimensional measures of ASD and ADHD and/or categorical diagnoses. We searched for empirical studies with respondents beyond age 12, and if papers reported on a clinical diagnosis of ADHD and/or ASD we checked if the age of onset criteria were (retrospectively) determined, in accordance with DSM-5 criteria. Electronic literature searches via PubMed and Psychinfo were conducted for articles that were published before October 2016 (unlimited by date of publication) in the English language (unlimited by date of publication). We used broad search terms to not miss out on relevant work, subsequently scrutinizing abstracts, and if needed the full text, to determine if the study reported on findings relevant in relation to developmental aspects of core symptoms and brain characteristics. We were particularly keen on finding studies with multiple measures over time to capture possible longitudinal change. We confined ourselves to studies bases on humans and filtered out those that were not described in English. Given that the information we were after is oftentimes “hidden” in papers (e.g., although not apparent from the title that a paper had a developmental focus, a correlation between age and symptoms could be provided), our research strategy was to employ a very lenient threshold at the level of inclusion based on the title, after which we made a more stringent selection of those paper that provided relevant developmental information. The following combinations of keywords were used to identify the relevant articles: ‘autism/ASD’, ‘ADHD’, ‘social’, ‘attention’, ‘activity’, ‘impulsivity’ ‘adolescence/adolescents’, ‘adult’, ‘longitudinal’, ‘prospective’, ‘development’, ‘brain’, ‘structural MRI’, ‘neuro’, and ‘structural imaging’. Both original research articles and meta-analyses, and systematic and narrative reviews were included, and references in these papers were checked for additional relevant literature. Despite these attempts, we do not claim to be exhaustive and cover all papers that may have met the search criteria. Rather we want to highlight the need for a lifespan-approach in studying ASD–ADHD co-occurrence in relation to structural brain imaging and discuss findings in the context of an overarching disorder hypothesis.

We will start with an overview on the normal developmental pattern of structural brain correlates and associated etiological factors, followed by the findings for ADHD, then ASD and—although scarcely available—by findings directly related to both. Findings will be summarized and in the discussion we elaborate on new insights in the light of the overarching disorder hypothesis.

## Results

### Brain correlates and underlying mechanisms of typical development across the lifespan

Recent years have seen much progress in our knowledge on life course, and in particular adolescent brain development. These normative changes may follow different patterns, and in transaction with the environment, have unique consequences for ASD and ADHD (Picci and Scherf 2015; Shaw et al. 2015). From childhood to young adulthood, several studies using magnetic resonance imaging (MRI) scans have shown developmental changes of white and gray matter volumes and overall brain volume (see for reviews Hedman et al. 2012; Lebel and Beaulieu 2011; Mills and Tamnes 2014). Around 9 years of age a 1% annual brain growth is found which levels off until at age 13 and a gradual volume decrease sets in. White matter volume significantly increases with age till around age 30, while there is a concomitant decrease in gray matter volume. In contrast to common views on brain development, between late adolescence and young adulthood (18–35 years) possibly another wave of growth occurs or at least a period of no brain tissue loss. After age 35 years volume loss sets in which accelerates after age 60 (Hedman et al. 2012).

With increasing age between birth and young adulthood, the brain is increasingly organized into distinct neural networks, with increasing integration within networks and increasing segregation between networks (Sherman et al. 2014). Subcortical regions mature earlier than prefrontal regions (involved in controlled cognition) (Mills et al. 2014). The maturation of the prefrontal cortex shows a predominantly linear trajectory until late adolescence/young adulthood, but subcortical structures like the striatum a quadratic trajectory, leading to a developmental disbalance of prefrontal control of subcortical functioning during adolescence (Casey et al. 2010; Galvan et al. 2006, 2007). This is possibly related to increased risk-taking, sensation-seeking behaviors and emotionality during adolescence, but also to increased ability for divergent thinking and creativity (Kleibeuker et al. 2013). Healthy cognitive aging appears to be linked to a preservation of structural brain integrity in old age rather than recruitment of ‘reserves’ (i.e., other brain networks) for maintaining cognitive abilities (reviewed in Draganski et al. 2013). This concept of ‘brain maintenance’ assumes the existence of a threshold of neurodegeneration burden, explaining superior cognitive performance by the preservation of the structural and functional integrity of the brain. In contrast, accelerated cognitive decline is mostly linked to neurodegeneration of structures in the prefrontal cortex (Henstridge et al. 2015).

It is unknown how these patterns of brain development across the lifespan are (dis)similar for males and females, since most MRI studies do not provide sufficient data to investigate differential effects between males and females across development (Hedman et al. 2012). Studies that do report on sex differences suggest brain volume may peak earlier in girls than in boys with a greater decline in cerebral volume in girls as compared with boys during adolescence (Lenroot et al. 2007; but see Ment et al. 2009). These sex differences on brain development shed light on the sex differences in normal adolescent development, but not on ASD and ADHD prevalence differences between the sexes. For that, more insight is needed in sex differences regarding very early brain development, from conception to school-age, the developmental period where the first precursors and symptoms of both disorders usually emerge. Nonetheless, they may aid explaining possible sex differences in the course of ADHD and ASD, a topic that has not been studied so far. Most studies in adulthood and old age suggest the rate of change in whole brain volume is similar in males and females (reviewed in Hedman et al. 2012).

When reviewing the underlying mechanisms responsible for these substantial brain changes during development, the heritability estimates of brain structures appear to change over the lifespan. Heritability estimates indicate how much variation in a trait in a population is due to genetic variation among individuals in that population. Heritability must be estimated from the similarities observed in subjects varying in their level of genetic or environmental similarity. Heritability estimates can change without any genetic change occurring, such as when the environment starts contributing to more (or less) variation; what matters is the relative contribution. It has been reported that brain areas that develop phylogenetically and ontologically early, such as the primary sensory cortex, show greater heritability *in childhood*, whereas brain areas more pronounced in humans and involved in cognitive functions predominantly found in higher functioning species (like the dorsal prefrontal cortex) show greater heritability *in adolescence* relative to childhood (Lenroot et al. 2009). A meta-analysis on the heritability of brain development reported that heritability is stronger for brain structure (60–80%) than function (~40%), although the latter estimate was based on relatively few studies conducted so far and is usually less reliably estimated (lower test–retest reliability) (see for review Jansen et al. 2015). Substantial heritabilities were found for cortical surface area, cortical thickness, gray matter volume, and white matter volume, although estimates differed dependent on the region investigated. Overall, during development from childhood to adulthood, heritability estimates for most volume measures of brain structures slightly increased, but strong differences were found between white and gray matter volumes, with high heritability estimates for white—but not gray—matter already from birth onwards, possibly suggesting virtually no environmental influences on the ‘hard wiring’ of the brain (Jansen et al. 2015). Heritability measures for white matter microstructure (measured using DTI) showed lower heritability estimates compared to volume measurements (White and Gottesman 2012). Overall, findings suggest that heritability estimates for most brain structures are somewhat higher in adult twin samples than childhood/adolescent twin samples, but conclusions regarding *developmental change* in genetic effects cannot be drawn due to the cross-sectional nature of the samples, the collapsing of broad age ranges in one single group, and the absence of studies including age as moderator in analyses. A longitudinal study including a large group of children, adolescents and adults found that the heritability of cortical thickness increases gradually throughout late childhood and adolescence (Schmitt et al. 2014). To what extent these genetic influences on the developing brain are different in ADHD or ASD is currently unknown. Yet this is plausible, given the findings of strong heritabilities in brain areas associated with cognitive control, which is strongly linked to ADHD and ASD.

Zooming in on sex differences regarding genetic effects on brain development is particularly relevant given that the prevalence of ASD and ADHD are known to differ between the sexes. Brain specific sex-biased gene expression may to some extent underpin this prevalence difference. A meta-analysis specifically examining the role of sex-chromosomes on brain development, concluded that although the X and Y chromosomes have opposing effects on overall brain size, they exert highly convergent influences on local brain anatomy across phylogenetically and ontologically differentially developing brain areas, particularly centers for social perception, communication, and decision-making (Raznahan et al. 2016), cognitive domains that play a key role in ASD and ADHD. In contrast, a meta-analysis examining the sex-biased expression of genes including all chromosomes in various human tissues, reported significant sex differences in gene expression in the brain, specifically in the anterior cingulate cortex (1818 genes) (Mayne et al. 2016). Surprisingly, many of these sex-biased genes were not under the direct influence of sex chromosome genes or sex hormones. These results combined suggest that sex-chromosomes appear not to play a major role in explaining sex differences regarding brain development (except overall size) and do not account for strong prevalence difference of ASD and ADHD between males and females; rather sex-biased expression of autosomal genes in the brain may account for sex differences in sexually dimorphic traits, among which ASD and ADHD.

The above described normative brain changes are not solely pre-programmed, but also under strong influence of a variety of environmental factors, such as (positive) parenting (Whittle et al. 2014), stressful life events (Luby et al. 2013; Lupien et al. 2009), or alcohol consumption (Luciana et al. 2013). The brain is all but static, strongly depending on the life phase specific alterations that occur within typical development and in transaction with the environment. There appear to be crucial time windows of plasticity that accommodate the new developmental tasks that individuals face (Khundrakpam et al. 2013, 2015). Suboptimal development during this sensitive time as this may be the case in ASD and ADHD may set the stage for changes in brain development later on. Understanding the mechanisms underlying these plastic brain changes—particularly during windows of increased plasticity—may contribute to distinguishing progressive brain changes in ADHD and ASD from healthy developmental processes.

### Brain correlates of ADHD across the lifespan

During the last two decades, a large number of imaging studies have documented on structural brain characteristics in children with ADHD (see for reviews Cortese et al. 2012; McCarthy et al. 2014; Kasparek et al. 2015; Philip et al. 2012; Via et al. 2011). Volumetric studies have generally reported decreased cortical thickness and gray matter volumes in adults with ADHD relative to healthy controls (Proal et al. 2011). Focusing on longitudinal repeated MRI studies, including adolescents and adults, compared to what is known on normative changes, a series of studies by Shaw and colleagues on individuals with ADHD and controls are of relevance. One of the initial studies reported that children with ADHD showed relative slow cortical thinning in regions important for attentional control between the ages of ~9 years and ~14.5 years (Shaw et al. 2006, Shaw et al. 2007a, b; later (also independently) replicated using dimensional measures of ADHD: Ducharme et al. 2012; Shaw et al. 2011). In addition, abnormalities in the development of cortical asymmetries from childhood (~10 years) to adolescent age (~17 years) were found (Shaw et al. 2009). This cohort has now been followed longitudinally until young adulthood (~24 years). The delay in cortical thinning was found to be related to a delay in the developmental trajectories of the surface area, but not in those of cortical gyrification (Shaw et al. Shaw et al. 2012a, b) and cortical thickening or minimal thinning was found exclusively among individuals who remitted (Shaw et al. 2013). Findings from this longitudinal sample thus suggest that delayed, and subsequently fixed cortical thinning may be a marker for ADHD persisting into adulthood.

Findings from other research groups are beginning to emerge. A cross-sectional comparison of cortical thickness in children, adolescents and adults with ADHD indicated that a thinning of the cortical surface in the right frontal lobe was present in all age groups (Almeida et al. 2010). However, an ongoing longitudinal pediatric neuroimaging study conducted across ages 5–25 years in both healthy and clinical populations, suggest white matter volumes increase and gray matter volumes follow an inverted U trajectory, with peak size occurring at different times in different regions. At a group level, differences related to ADHD are seen for gray and white matter volumes, rates of change, and for interconnectedness among disparate brain regions, illustrating the complex mix of ADHD brain correlates across age (Giedd et al. 2015).

When zooming in on MRI studies in adolescents with ADHD, an abnormally high fractional anisotropy (FA) in frontal networks, smaller prefrontal volumes, decreased orbitofrontal volumes and abnormalities in insula, occipital, and somatosensory areas have been documented.(Davenport et al. 2010; Francx et al. 2016). Evidence for differential brain maturation processes underlying symptom domain specific improvements during adolescence has also been reported: lower FA and mean diffusivity (MD) (indices of white matter microstructure) in the left corticospinal tract at follow-up were associated with improvements of hyperactive/impulsive—but not inattentive—symptoms. Since the corticospinal tract is important in the control of voluntary movements, this may pinpoint to differential brain mechanisms underlying developmental changes during adolescence in the various symptom domains (Francx et al. 2015). In addition, abnormalities in subcortical volumes like the basal ganglia have been reported in adolescents with ADHD (and their first degree non-affected relatives) (Greven et al. 2015; Sobel et al. 2010), with cross-sectional findings suggesting these volumes decrease with age in controls but not in participants with ADHD (Greven et al. 2015). In a longitudinal imaging study following children (~10.5 years) until early adolescence (~13 years), a nonprogressive loss of volume was reported in participants with ADHD in the superior cerebellar vermis which did not correlate with outcome. In contrast, progressively decreasing inferior-posterior cerebellar lobes correlated with worse outcome (but note, participants were still in early adolescence) (Mackie et al. 2007). These findings indicate significant age moderating effects occur during adolescence, possibly explaining the significant change in ADHD symptoms that take place in a proportion of patients during this time window.

A review on imaging studies in children and adults with ADHD concluded that results pointed to the persistence of changes in brain structure into adulthood, although there might be a tendency for improvement of caudate nucleus pathology (Kasparek et al. 2015). Furthermore, cross-sectional MRI studies in adults have reported no differences between persistent and remittent ADHD as well as region specific increased thickness in patients with ADHD (Duerden et al. 2012; Proal et al. 2011). In a cross-sectional population-based sample of adults in their late 60s and early 70s, it was examined whether ADHD symptoms correlate with callosal thickness (Luders et al. 2016). Results indicated that there were negative correlations between several regions within the corpus callosum and ADHD symptoms, possibly suggesting reduced inter-hemispheric communication/myelination related to ADHD symptoms at this age. However, these findings only pertained to males. In females, opposite findings emerged with callosal thickness (rostral body) being positively related to hyperactivity. The possibility of sexually dimorphic neurobiology of ADHD symptoms at this age as well as earlier in development remains a real possibility (Cortese et al. 2012; McCarthy et al. 2014; Philip et al. 2012; Via et al. 2011).

With a growing number of publications by independent research groups on longitudinal brain measures collected in individuals with ADHD, it is expected that in the upcoming years a more solid view will emerge on brain imaging correlates of persistent and remitted (or even adulthood-onset; see Agnew-Blais et al. 2016; Caye et al. 2016; Moffitt et al. 2015) cases of ADHD in males and females.

### Brain correlates of ASD across the lifespan

Relative to ADHD, longitudinal MRI studies in adolescents and adults with ASD are scarce. Several lines of research suggest that cortical development in ASD sets out with accelerated expansion in early childhood, accelerated thinning later in childhood, and atypical thinning (decelerated versus accelerated, depending on cortical region) in early adulthood (Courchesne et al. 2011; Lange et al. 2015; Ismail et al. 2016; Sacco et al. 2015; Wallace et al. 2015; Zielinski et al. 2014). This atypical trajectory of brain maturation gives rise to differences in functioning and connectivity and may make individuals with ASD at risk for accelerated cortical decline in later life (Ecker et al. 2015). Importantly, cortical thickness abnormalities in ASD are region-specific and remain dynamic well into adulthood (Ismail et al. 2016; Lange et al. 2015; Wallace et al. 2015; Zielinski et al. 2014; but see Wallace et al. 2012, reporting on developmentally stable thinner right superior temporal sulcus in relation to subclinical ASD traits in typically developing children and adolescents). Cross-sectional attempts to map age-related changes into adulthood confirm moderating effects of age on brain anatomy in ASD (e.g., Ismail et al. 2016; Itahashi et al. 2014; Kleinhans et al. 2012; Saitoh et al. 2001; Schumann et al. 2004), with potentially nonlinear relationships between subcortical volumes and brain volumes inducing heterogeneous findings in different study samples (Lefebvre et al. 2015). A recent meta-analysis on brain structures involved in social cognition documented significant increases in cortical thickness in individuals with ASD in the left pars opercularis aspect of the inferior frontal gyrus relative to age, and to mean thickness of the left hemisphere (Patriquin et al. 2016). Furthermore, interactions examining the combined effect of diagnosis, age and mean thickness revealed that the thickness of the left pars opercularis decreased in individuals with ASD as a function of age and as a function of the mean thickness of the right hemisphere combined. A similar effect was also noticed in the right fusiform gyrus, with ASD-control group differences heavily influenced by the interactions between age and mean thickness of the right hemisphere (Patriquin et al. 2016). Significant age moderating effects on subcortical structures in ASD have also been reported for the amygdala and the cerebellar vermal lobules VI-VII (Sacco et al. 2015; Stanfield et al. 2008). Despite the cross-sectional nature of these studies, age moderating effects on ASD-control group differences are clearly present and deserve attention within longitudinal studies.

### Overlapping brain correlates of ADHD/ASD across the lifespan

Several imaging studies have compared brain characteristics in adolescents/young adults with ASD and ADHD symptoms (Brieber et al. 2007; Christakou et al. 2013; Fine et al. 2014; Lim et al. 2013, 2015). Findings are heterogeneous, with overlapping and nonoverlapping findings related to ASD and ADHD, but no consistent pattern has so far emerged. One study showed gray matter *reductions* in the medial temporal lobe and gray matter *increases* in the left inferior parietal cortex in *both ASD and ADHD* in adolescence, with somewhat more profound increased gray matter volume in the right supramarginal gyrus in adolescents with ASD (Brieber et al. 2007). Other studies reported on dissimilar brain characteristics associated with ASD and ADHD in adolescence/adulthood. In one study, it was reported that adolescent boys (11–17 years) with ADHD showed gray matter *reductions* of the right posterior cerebellum and left middle/superior temporal gyrus, whereas adolescent boys with ASD showed to a lesser extent gray matter *increases* in the left middle/superior temporal gyrus (Lim et al. 2013, 2015). A small study comparing corpus callosum volumes in young adolescents [~13 years (8–18 years)] with ASD and ADHD, reported that larger midbody areas compared to controls were found in adolescents with ASD but not ADHD (Fine et al. 2014). Quantitative measures of ASD and ADHD in relation to gray matter volumes in healthy young adults (18–29 years) also showed non-overlapping results, with ASD symptoms correlating with the left posterior cingulate, and ADHD symptoms with the right parietal lobe, right temporal frontal cortex, bilateral thalamus, and left hippocampus/amygdala complex (Geurts et al. 2013, but see Koolschijn et al. 2015 for non-replication of ASD findings). This heterogeneous mix of findings is difficult to interpret due to the lack of longitudinal brain measures used, the small sample sizes with large heterogeneity within the clinical and control groups, the often arbitrary distinction between both clinical groups where comorbid ADHD is largely ignored in ASD studies and high levels of ASD symptoms are present in subjects with ADHD (as reported by for instance Brieber et al. 2007, the ASD affected group had equally high levels of ADHD symptoms as the ADHD affected group) and the various methods used to select brain measures of interest.

As indicated, no studies have longitudinally and simultaneously mapped ASD and ADHD symptoms in relation to brain characteristics in adolescents or adults. However, one longitudinal imaging study examined effortful control (self-report) in adolescents (T1 ~13 years, T2 ~16.5 years). Effortful control—the ability to voluntarily manage attention and inhibit or activate behavior as needed to adapt, especially in the context of low motivation—is associated with both ASD and ADHD. A study in healthy control participants reported that a greater age-related thinning of the left anterior cingulate cortex was associated with less reduction in effortful control and in turn with improvements in socio-emotional functioning and reductions of psychopathological symptoms (aggression, mood and anxiety) (Vijayakumar et al. 2014). In other words, an increased/accelerated thinning of the anterior cingulate cortex was associated with more stable levels of effortful control and in turn with better socio-emotional functioning. Although no direct link with ADHD or ASD was made in this study, the findings correspond with the studies reporting on delayed/decelerated thinning in adolescence/young adulthood in ASD and ADHD. In a cross-sectional attempt to map gray and white matter volume changes in relation to both ADHD and ASD symptoms in ADHD affected adolescents [~17 years (10–26 years)] (O’Dwyer et al. 2014, 2016), ASD symptoms strongly diminished with age in ADHD affected adolescents. Subjects with ADHD without ASD symptoms had smaller total brain volumes than control subjects, which was not the case for adolescents with ADHD and stable high ASD symptom levels. Subclinical elevated ASD symptom levels were associated with more gray matter volume, whereas more clinical levels of ASD symptoms were associated with both increased gray and white matter volumes (O’Dwyer et al. 2014). Furthermore, the caudate nucleus and globus pallidus volumes appeared of critical importance in predicting the level of ASD-like symptoms of participants with ADHD (O’Dwyer et al. 2016). However, these effects did not appear to be related to age, albeit the findings pertained to individuals of different ages rather than longitudinal changes.

## Summary and discussion

In summary, in healthy controls distinct developmental trajectories have been observed for cortical thickness, myelination and maturation of distinct neural networks, volume changes in individual structures, and hemispheric specialization. Cortical thickness can be used to estimate individuals’ brain maturity. What is clearly evident is that brain maturation in ADHD and ASD is altered or delayed from (early) childhood on. This likely changes the brain further on in development, compounding effects due to altered daily life experiences and compensatory brain changes. That the typically developing human brain is not fully mature until well into adulthood—and also remains to a certain degree receptive and flexible during the entire life—suggests that in ASD and ADHD cortical maturation is likely protracted until further across the lifespan, in combination with permanent alterations. The vast majority of existing MRI studies, however, has been cross-sectional, based on small samples and conducted in children, and sometimes did include adolescents as well, but without explicitly documenting on this age group. MRI studies documenting on age effects in adults with ASD and/or ADHD are rare, and if age is taken into account, only linear effects are examined. The few studies directly comparing brain characteristics associated with ASD and ADHD provide a heterogeneous mix of findings that is difficult to interpret due to the lack of longitudinal brain measures, the various methods used, and the sometimes arbitrary distinction between both clinical groups.

ADHD and ASD symptoms are developmentally complex phenotypes characterized by both continuity and change across the life span. We plotted six hypothetical developmental ASD–ADHD subtypes based on the overarching disorder hypothesis (see Fig. 1). We hypothesize that these different developmental subtypes may have distinct causes and distinct functional outcomes. A crucial distinctive feature underlying these various developmental subtypes may be the differential developmental thinning patterns of the anterior cingulate cortex and related connections towards other prefrontal regions. As reviewed above, these regions are crucial for the development of cognitive/effortful control and poorer socio-emotional functioning (Alahyane et al. 2014; Cachia et al. 2016; Gariépy et al. 2014; Lincoln and Hooker 2014; Ordaz et al. 2013; Peters et al. 2016), two key common features underlying both ASD and ADHD. Currently, data are lacking to confirm or refute these ASD–ADHD developmental subtypes as well as the potential role of distinct developmental thinning patterns of the ACC and related connections towards other prefrontal regions underlying these developmental subtypes. Longitudinal studies are needed that repeatedly map both ASD and ADHD symptoms from childhood age onwards until late adulthood and relate these symptom trajectories to repeatedly measured brain characteristics.

Our review indicates that findings on neural mechanisms underlying ASD and ADHD in childhood years cannot be automatically extrapolated to adolescence and adulthood. Findings from genetic factors underlying brain structure show greater heritability in childhood for earlier maturing areas and greater heritability in adulthood for later maturing areas. Understanding the mechanisms underlying these normative brain changes—particularly during windows of increased plasticity—may contribute to distinguishing progressive brain changes in ADHD and ASD from healthy developmental processes. It is likely that adolescence and young adulthood are crucial phases wherein new genetic effects may become relevant in determining further development of cortical thickness, general cognitive ability and potentially neurodevelopmental disorders (Greven et al. 2011; Pingault et al. 2015). Suboptimal brain development during this sensitive time as this is the case in ASD and ADHD may set the stage for significant changes in brain development later on. Available evidence suggests these genetic factors influencing suboptimal brain development are largely generic instead of disorder-specific.

The clear age effects on—and large within group heterogeneity of—brain anatomy in relation to ASD and ADHD symptoms, do call into question the attempts to design uniform brain-based diagnostic classification algorithms that largely ignore these age effects and within group heterogeneity (see for extensive reviews Arbabshirani et al. 2016; Wolfers et al. 2015). We believe that developmentally and gender sensitive MRI markers need to be used to detect biologically more homogeneous subtypes. Needed are empirical modeling approaches that go beyond ‘lumping and splitting’ (see for review Marquand et al. 2016b) to re(de)fine developmental phenotypic subgroups based on multidimensional, longitudinally measured quantitative symptom levels instead of using cross-sectional, DSM-based heterogeneous groups. Given the tremendous methodological progress that is being made in the field neuroimaging (like multimodal MRI), it becomes increasingly possible to link multivariate neurobiological mechanisms to these behaviorally refined subtypes, for instance using a normative modeling approach (Marquand et al. 2016a). Normative modeling provides a natural framework to estimate the deviation from normal functioning at the individual level without dichotomizing the cohort. Such efforts are needed to be able to detect etiologically distinct developmental subtypes, which in turn can inform prognosis and choice for intervention.
